# Adult tissue-specific stem cell interaction: novel technologies and research advances

**DOI:** 10.3389/fcell.2023.1220694

**Published:** 2023-09-21

**Authors:** Xutao Luo, Ziyi Liu, Ruoshi Xu

**Affiliations:** State Key Laboratory of Oral Diseases, National Center for Stomatology, National Clinical Research Center for Oral Diseases, Department of Cariology and Endodontics, West China Hospital of Stomatology, Sichuan University, Chengdu, China

**Keywords:** stem cell interactions, dual lineage tracing, synthetic receptor, transcriptomics, proximity labeling

## Abstract

Adult tissue-specific stem cells play a dominant role in tissue homeostasis and regeneration. Various *in vivo* markers of adult tissue-specific stem cells have been increasingly reported by lineage tracing in genetic mouse models, indicating that marked cells differentiation is crucial during homeostasis and regeneration. How adult tissue-specific stem cells with indicated markers contact the adjacent lineage with indicated markers is of significance to be studied. Novel methods bring future findings. Recent advances in lineage tracing, synthetic receptor systems, proximity labeling, and transcriptomics have enabled easier and more accurate cell behavior visualization and qualitative and quantitative analysis of cell-cell interactions than ever before. These technological innovations have prompted researchers to re-evaluate previous experimental results, providing increasingly compelling experimental results for understanding the mechanisms of cell-cell interactions. This review aimed to describe the recent methodological advances of dual enzyme lineage tracing system, the synthetic receptor system, proximity labeling, single-cell RNA sequencing and spatial transcriptomics in the study of adult tissue-specific stem cells interactions. An enhanced understanding of the mechanisms of adult tissue-specific stem cells interaction is important for tissue regeneration and maintenance of homeostasis in organisms.

## Graphical Abstract

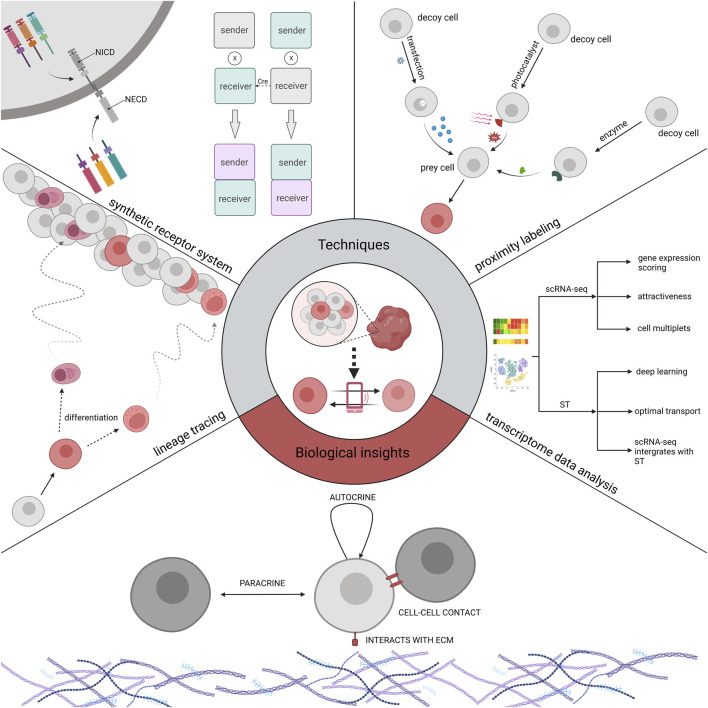

## 1 Introduction

Adult tissue-specific stem cells interactions can be viewed as communication modalities that play a central role in regulating cell behavior and function (Xin et al., 2016).

In this paper, we first discuss the methods for studying adult tissue-specific stem cells interactions in two directions: bioinformatics analysis and visualization analysis. Specifically, these techniques or methods include lineage tracing, synthetic receptor systems, proximity labeling, and transcriptome analysis. In addition, there are few systematic descriptions of the mechanisms of stem cell interactions. Therefore, this paper also combs the biological understanding of adult tissue-specific stem cells interactions. We do not provide a summary assessment of all relevant literature, and references throughout the text tend to be more illuminating examples. Based on the latest literatures, stems cells referred in this review focus on adult tissue-specific stem cells.

## 2 Tracking the targets all the way: lineage tracing

**FIGURE 1:**
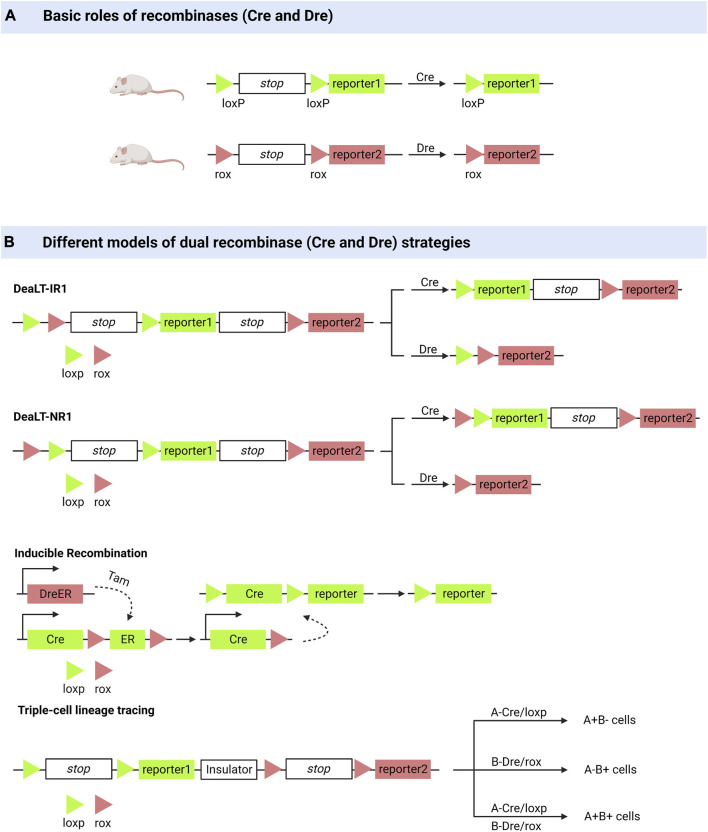
Dual enzyme lineage tracing system. **(A)** Cre or Dre recombinases can knock out sequences between loxP or between rox. **(B)** DeaLT-IR: the loxp and rox sites are arranged alternately in a sequence. DeaLT-NR: two sites of loxp are contained within two sites of rox. Inducible Recombination: the DreER was activated with the inducer tamoxifen to produce a Dre recombinase to remove the sequence between the two rox sites. Triple-cell lineage tracing (an example): On a sequence, two sites of loxp and their corresponding reporters were distributed on either side of an insulating sequence, and cells expressing different recombinant enzymes (Cre, Dre, Cre and Dre) showed three reported results.

Lineage tracing is a powerful means of monitoring cells in various physiological and pathological processes, resulting in many valuable biological discoveries (Kumar et al., 2014; He et al., 2017a; Wu et al., 2021; Hou et al., 2022). Cell lineage tracing techniques have used classical fluorescent protein markers combined with microscopy to identify a few cell clones and to introduce heritable DNA barcodes into single cells to track larger numbers of more complex cell clones, the latter including Cre-mediated recombination and CRISPR-Cas9-mediated editing (Meinke et al., 2016; Baron and van Oudenaarden, 2019). Optogenetic engineering has been developed to some extent. It has been used for detailed *in vivo* single-cell analysis because of its efficiency and spatiotemporal specificity (Yao et al., 2020; Li et al., 2022a; Geiller et al., 2022). Here, we describe the advances in cell lineage tracing technology, with a focus on Cre/LoxP recombinase systems (Figure 1).

### 2.1 Genetic lineage tracing has entered the era of the recombinase system

The Cre/LoxP recombinase system is widely used for *in vivo* tracking of stem or progenitor cell lines. Its main working principle can be briefly summarised as follows: Cre mediates the removal of transcription termination DNA sequences on the LoxP side, whereupon predesigned reporter genes are expressed in cells expressing Cre (Meinke et al., 2016). Coupled with the fact that this genetic marker can be passed on to progeny cells, this feature allows researchers to elucidate cell lineages as well as track cell fate decisions in stem or progenitor cell lines (van Berlo et al., 2014; Liu et al., 2016; Zhu et al., 2016). However, many controversies have arisen regarding the drawbacks of the Cre/LLoxP system (Liu et al., 2016). Single-spectrum tracing has been criticized for a long history (van Berlo et al., 2014). The complexity of Cre transgene expression patterns and the toxicity generated to activate Cre are of concern, and in some cases, the data obtained by applying the Cre/LoxP knock-in system have been proven incorrect (Zhu et al., 2016; Pu et al., 2018). He et al. (2017a) pioneered the Cre: Dre dual-recombinase reporter system. This system combines the Cre/LoxP system with the Dre/rox system, where the Dre/rox recombination system releases Cre from the CreER and allows Cre targeting of the LoxP allele. Determining Cre activity using two different gene promoters provides a higher gene targeting precision than was previously possible with a single gene promoter controlling Cre. Besides being used for cell lineage tracing, it can also be used to query the functions of cell subpopulations *in vivo* (Pu et al., 2018).

#### 2.1.1 Improved spatial resolution

The ability of bronchioalveolar stem cells (BASCs) to regenerate the bronchoalveolar junction region after an injury has been demonstrated using dual-recombinase-activated lineage tracing (DeaLT) system, reiterating previous findings (Salwig et al., 2019; Liu et al., 2020a). Applying the same technique, one demonstrated the dominance of self-renewal of pancreatic β-cells in pancreatic β-cell regeneration (Zhao et al., 2021). The idea that pancreatic progenitor cells give rise to pancreatic β-cells was challenged. DeaLT has also been applied to bone tissues. The division of labor between chondrocytes and Lepr^+^ bone marrow stem cells (BMSCs) during long bone formation is clearly illustrated (Shu et al., 2021). It has also been revealed that a subpopulation of Krt14+ Ctsk+ cells involved in maxillary bone regeneration has both epithelial and mesenchymal properties (Weng et al., 2022). In lung and liver injury, it was previously thought that macrophages that are non-vascularly recruited to reach the viscera via CD44 and ATP directly promote the repair and regeneration of the injured viscera (Wang and Kubes, 2016; Deniset et al., 2019). Updated techniques have helped re-evaluate this idea, and the results of the experiments do not support the scientific validity of the idea (Jin et al., 2021a).

#### 2.1.2 The time continuity of lineage tracing is realized

Based on the Cre: Dre dual recombinase reporter system, Lingjuan He et al. (2021) developed a cell proliferation tracer model, called “ProTracer.” Specifically, DreER-rox recombination removes the estrogen receptor DNA on the rox side, whereupon the sequence encoding CrexER is converted into constitutively active Cre gene sequences, resulting in cells of Ki67-Cre genotype. In these cells, the expression of constitutively active Cre gene sequences is triggered by the Ki67 promoter, and the transcriptional activity of Ki67 is continuously recorded by the R26-GFP reporter gene activation. This model allows the tracing of Ki67+ cells at any moment and the recording of cell proliferation over time. ProTracer combines dual recombinases to continuously document the proliferation of whole cell populations in multiple tissues and organs, thereby eliminating potential selectivity bias. The use of ProTracer allows for the sequential recording of the initial tamoxifen-triggered DreER-rox recombination initiating the Ki67- Cre gene, thus overcoming the technical dilemma of using Cre/LoxP alone, which requires prolonged CreER activation with tamoxifen (He et al., 2021). ProTracer records circulating cardiomyocytes in both dividing and non-dividing cardiomyocytes. Most circulating cardiomyocytes and dividing cardiomyocytes (approximately 13% of tracer cardiomyocytes) are highly confined to the subendocardial muscle of the left ventricle of the adult heart (Liu et al., 2021). They also assessed postnatal cardiomyocyte proliferation using ProTracer, which showed a rapid and sustained decline in the number of circulating cardiomyocytes from birth to adolescence. These results support the claim that prepubertal cardiac proliferation shows a burst (Pu et al., 2022).

### 2.2 Optogenetics has a promising future in genetic lineage tracing

We also note the contribution of optogenetic genetic engineering to the content of our work, which is widely used to study and control cells and has been previously described (Tan et al., 2022). Optogenetics regulates cell physiology by combining light with genetic engineering. The advantages of high spatiotemporal accuracy and single-cell resolution offered by optogenetic tools have made it a powerful technique for the precise detection of signaling and intercellular interactions (Leopold et al., 2018; Uroz et al., 2018; Gagliardi et al., 2021; Zhang et al., 2021; Kim and Schnitzer, 2022). Felker et al. (2018) described the formation of zebrafish ventricles by progenitor cells using optogenetic techniques for lineage tracing. The combination of optogenetic genetic engineering with Cre/LoxP systems has also been experimentally implemented (Taslimi et al., 2016; Morikawa et al., 2020); however, its limitations are related to the lack of specificity of the Cre/LoxP system itself (Tan et al., 2022). Optogenetic tools provide a tunable platform for studying the dose- and intensity-dependent signaling between sending cells and receiving cells (Tan et al., 2022). Moreover, current research models in optogenetics are not sufficiently rich, and we suggest that optogenetic genetic engineering can be applied to study the function of adult tissue-specific stem cells in homeostasis and regeneration. A combination of dual recombinant enzyme lineage tracer strategies and optogenetic engineering can be widely applied.

## 3 Customized cell signaling: synthetic receptor systems

**FIGURE 2:**
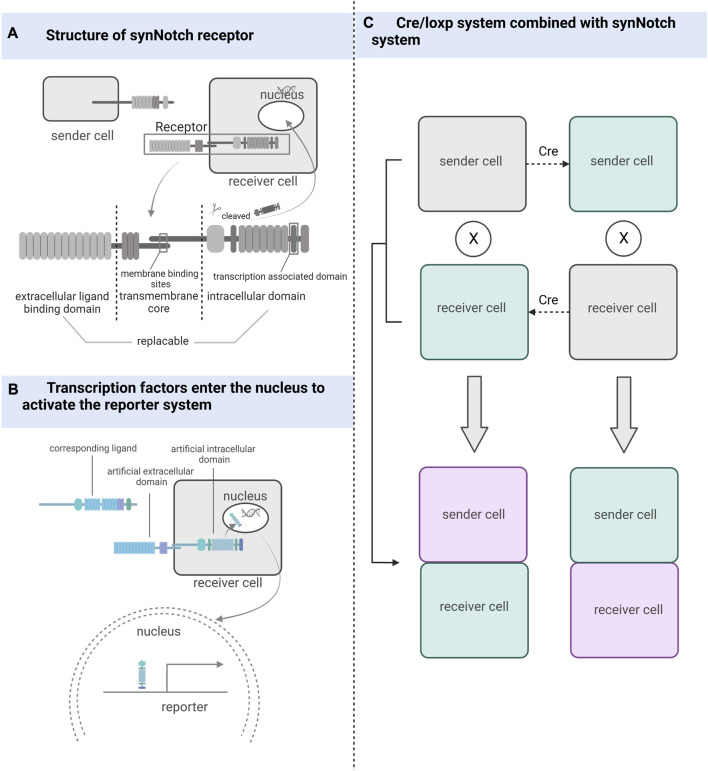
The working mechanism of the synthetic receptor system. **(A)** synNotch receptor consists of an extracellular ligand-binding domain, a native Notch receptor transmembrane core, and an intracellular domain linked to the transcriptional factor-associated intracellular domain. **(B)** After the ligand binds to the synthetic receptor, transcription factors located in the intracellular segment are cleaved and take effect in the nucleus. **(C)** Combining the Cre/loxP system with the synthetic receptor system can also be used to detect and track cells that have undergone cell-cell contact, whether sending or receiving cells.

Inspired by the natural cell-signaling paradigm, researchers have implemented artificially designed synthetic receptor systems to manipulate signal translation to control cellular functions, including artificially tunable cellular sensing and subsequent transcriptional responses (Zhu et al., 2022; Roybal et al., 2016; Morsut et al., 2016; Hernandez-Lopez et al., 2021). The development of synthetic receptor systems has gradually evolved from initial modest modifications of natural proteins to the holistic design of modular technologies that increasingly approach the engineering of customized cellular functions (Manhas et al., 2022). Artificial effects on cellular activity through synthetic receptors include but are not limited to, control of pluripotent stem cells differentiation (Lee et al., 2023). And if the endpoint of intracellular signaling is set to the expression of fluorescent genes, we can clearly distinguish which cells receive the influence of the sender cells (Figure 2).

### 3.1 Monitoring cell-cell contacts

The synNotch receptor system, which detects cell-cell contact, was developed during the development of synthetic receptor systems (Morsut et al., 2016). The synNotch receptor is described as a receptor that signals through intramembrane protein hydrolysis and contains an extracellular ligand-binding domain, a native Notch receptor transmembrane core, and an intracellular domain linked to transcriptional factor-associated intracellular domain (Toda et al., 2018). Both the extracellular and intracellular domains of the Notch receptor are artificially substitutable so that highly diverse cell-cell interactions can be artificially engineered (Struhl and Adachi, 1998; Gordon et al., 2015). The synNotch receptor system can potentially become a powerful tool for studying cell-cell contact by being designed in a rational way that exploits the precise spatial control of synNotch pathway activation dependent on direct cell-cell contact (Morsut et al., 2016).

### 3.2 Combination of synNotch with different reporting systems

Firstly, Huang et al. used different enhancers to drive synNotch ligands and receptors to monitor neuronal and glial cell interactions and achieve long-term genetic modifications in a *Drosophila* model (Huang et al., 2016). He et al. (2017b) improved this idea by combining the synNotch receptor with a transcriptional stop signal and flip-flopping enzyme (FLP) recombination target sites on either side. These sequences were placed before the GFP ligand and controlled using the same promoter. The removal of gene sequences between FLP recombination target sites was achieved using FLP recombination, and the GFP ligand was expressed only in the presence of FLP, enabling the permanent labeling of cells for lineage tracing and real-time capture of intercellular contacts. This attempt led to breakthroughs in mammalian cell models, where Toda et al. (2018) used synNotch to design a modular signaling platform to enable the operation of artificial genetic programs at the tissue level. Specifically, the team used specific cell-cell contacts to induce changes in calmodulin adhesion that altered the local signals received by the cells, demonstrating the potential of artificially manipulated tissue generation. Zhang et al. (2022a) used Myc-tagged anti-GFP nanosomes (αGFP) and tetracycline (tet) trans-activator (tTA) to replace the extracellular and intracellular domains in the synNotch receptor system, respectively, and designed a genetic marker for labeling cell-cell contacts using tetO-LacZ as a reporter allele and membrane-bolted green fluorescent protein (mGFP) as a ligand. The contact of the sending cell containing the ligand with the cell containing the corresponding synNotch receptor triggers cleavage of the Notch transmembrane structural domain, releasing tTA into the nucleus, which in turn activates the reporter allele (Zhang et al., 2022a). If the Cre/LoxP system is used instead of the tetO-LacZ reporter system, it is not only possible to determine whether cell contact has occurred but also to permanently track cells that have made cell-cell contact and their progeny. SynNotch, combined with the Cre/LoxP system, can be used as a tool for genetic tracing of cell-cell contact (Zhang et al., 2022a).

## 4 Proximity labeling

**FIGURE 3:**
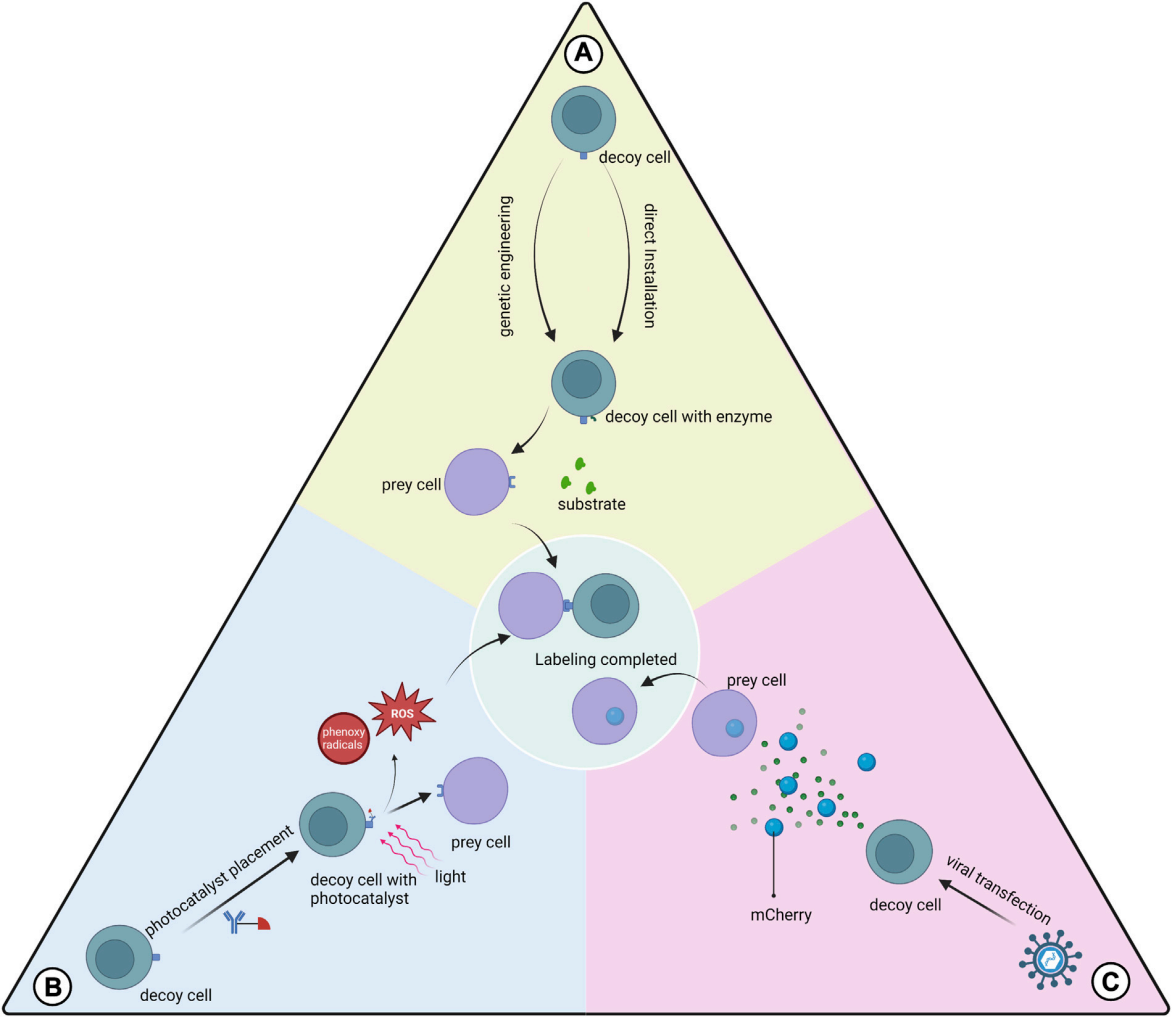
Three types of proximity labeling. **(A)** Enzymes are expressed on the cell membrane of the decoy cell by genetic engineering or are installed on the decoy cell by mediation such as antibodies to catalyze substrate labeling of the prey cell; **(B)** Photocatalysts pre-installed on the decoy cell are activated using light of a certain wavelength to generate free radicals at the interaction interface, which mediates the labeling; **(C)** Decoy cells transfected with viruses express and secrete markers that can be absorbed by the prey cell.

Many proximity labeling systems have been developed to analyze protein-protein interactions, but relatively few have been applied to study cell-cell interactions (Liu et al., 2020b). Substances with catalytic effects (enzymes or photocatalysts) are bound to membrane proteins of the target cell for the production of intermediates (Ge et al., 2019; Liu et al., 2022a). The added substrate is attached by the intermediate to the cell that interacts with the target cell. This process is called proximity labeling. In certain aspects, proximity labeling offers many advantages over other techniques for studying cellular interactions. The proximity labeling approach is closer to the natural environment than synthetic receptors. The data provided by bioinformatics functions primarily as a reference, and predictions made about cellular interactions require further validation. Techniques based on imaging to study cellular interactions do not provide molecular information about cellular interactions and do not isolate the cells of interest for subsequent analysis. According to our objectives, the limitation of the present proximity labeling method is that the cells studied are mostly immune cells and tumor cells, while few descriptions involving adult tissue-specific stem cells are available. We need to further validate whether proximity labeling is applicable to stem cell studies. Proximity labeling techniques commonly used for cellular interaction studies currently include both enzyme-based and photocatalyst-based types (Figure 3).

### 4.1 Enzymatic labeling

#### 4.1.1 Genetic manipulation

This class of proximity labeling protocols uses genetic engineering to make decoy cells express the enzymes used for proximity labeling and transfer label to prey cells. The main types of proximity marker technologies used to study cellular interactions are LIPSTIC, EXCELL and PUP-IT.

LIPSTIC uses sortase A (SrtA) genetically engineered to be expressed in decoy cells to transfer the markers to prey cells (Pasqual et al., 2018). This label can only be transferred when the cells are close enough to each other for the ligand receptor to bind to each other. This scheme provides information on the cells where the interaction occurs and quantifies this interaction. However, prior glycine placement of cells is required for the application of LIPSTIC. Recently the team described a modified version of LIPSTIC, uLIPSTIC, which is expected to enable the detection and analysis of CCIs across multiple organs (Nakandakari-Higa, 2023).In the EXCELL protocol, SrtA was improved to a variant mgSrtA, which has advantages in terms of cell labeling efficiency and signal-to-noise ratio. Compared to LIPSTIC, EXCELL does not require prepositioned glycine and also has the ability to discover unknown cell-cell interactions (Ge et al., 2019).The innovative feature of PUP-IT versus other neighboring labeling protocols is that both enzyme and substrate are proteins, and thus have better selectivity in genetic fusion strategies (Liu et al., 2018). PUP-IT2 is characterized by the minimization of fusion proteins and the virtual absence of self-labeling of enzyme (Yue et al., 2022). The relatively low labeling efficiency of the PUP-IT protocol has been reported in the literature and may not be suitable for studying cell-cell interactions (Ge et al., 2019).

#### 4.1.2 Non-genetic manipulation

The FucoID protocol installs the glycosyltransferase directly onto the decoy cell without gene expression on the cell membrane (Liu et al., 2020b). This enzyme allows the transfer of GDP-Fuc-GF-Biotin substrate to the prey cell, thus enabling proximity labeling. The development team used FucoID to detect and isolate T cells interacting with dentritic cells in a pancreatic tumor model with new bystander T cells, driving personalized cancer therapy. The team updated the tool library of the FucoID protocol and proposed two probes, cell-sFT and Ab-sFT (Qiu et al., 2022). The former requires pre-placement of fucosyltransferase (FT) on decoy cells, while the latter does not, thus it can be applied in cases where decoy cells are difficult to isolate.

### 4.2 Photocatalytic labeling

Protocols using photocatalysts have greater temporal control and remote manipulation than enzymatic proximity-labeling systems (Liu et al., 2022a). These protocols typically generate reaction intermediates such as free radicals at the cell-cell interaction interface to label prey cells.

PhoXCELL is an update of the EXCELL protocol (Liu et al., 2022a). PhoXCELL introduces Dibromofluorescein as a photocatalyst that mediates the transfer of cellular labeling using singlet oxygen (radicals). The diffusion radius of oxygen radicals is smaller compared to other radicals, reducing the possibility of false positive assay results. The PhoTag protocol uses phenoxy radicals to label neighboring cells (Oslund et al., 2022). This protocol recognizes and couples to decoy cell membrane proteins via an antibody-mediated photocatalyst and is primarily used to analyze physical interactions. This study also describes the combination of PhoTag with multi-omics single-cell sequencing to provide more information about the cells involved in the interaction. μMap uses carbene as a reaction intermediate, which has a shorter half-life compared to phenoxy radicals, which contributes to the resolution (Geri et al., 2020). μMap-red uses longer wavelength red light combined with a photocatalyst to generate from aryl azides nitrogen-containing radicals, which is suitable for *in situ* studies of cellular interactions in animal models (Buksh et al., 2022).

### 4.3 Direct labeling

We also focused on the mCherry-niche labeling system (Ombrato et al., 2021). This is a labeling method for detecting cells in the tumor microenvironment that receive secreted proteins from tumor cells (O et al., 2019). Through viral transfection, tumor cells stably express mCherry, and the stronger the tumor cell secretion capacity, the more mCherry is secreted. mCherry is readily taken up by other cell types and labels the cells. Further validation of the contribution of the mCherry-niche system to the study of the paracrine secretome of MSCs is needed.

### 4.4 Engineered virus labeling

Engineered viral labeling in fact goes beyond the definition of proximity labeling that we presented at the beginning of this section. However, the brief logic of engineered virus labeling is applying the ligand of interest to discover the receptor that interacts with it, which is consistent with the core idea of proximity labeling to discover prey cells that interact with decoy cells. Specifically, engineered viral labeling uses lentiviruses with artificially defined ligands to engage in ligand-receptor interactions with cells that have the corresponding receptors (Yu et al., 2022a). The interacting cells endocytose the corresponding engineered viruses. Cells are labeled by the barcode of the virus, thus allowing the identification of cells interact with that ligand. In addition, when multiple engineered viruses with different ligands with barcodes are used to mix with cells of interest, it is also possible to identify which ligands the cells interact with (Yu et al., 2022a).

## 5 Integration and systematization of transcriptomics analysis

**FIGURE 4:**
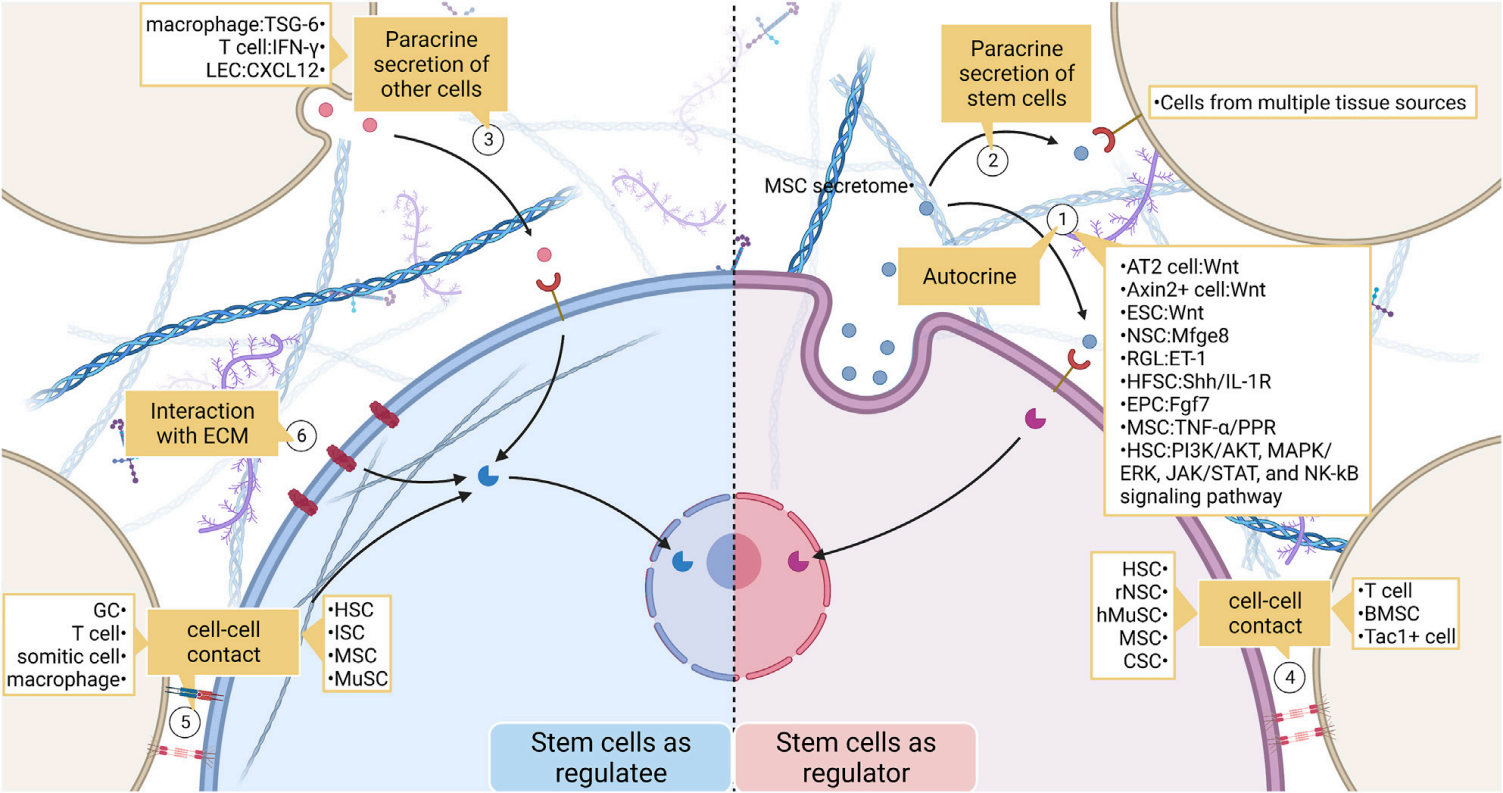
The broad landscape of stem cell interactions. (1) Stem cells act as signal senders and regulate their behavior. Effects include recruiting stem cells, maintaining the normal fate of stem cells, and influencing stem cell proliferation and differentiation. (2) (3) Stem cells communicate with non-stem cells by secreting chemicals. (4) (5) Stem cells and non-stem cells transmit mechanical or biochemical information to each other through cell-cell contact. (6) Stem cells also communicate closely with ECM. ECM regulates stem cell behavior by enriching ligands as well as by their physical properties. LEC, lymphatic endothelial cell; MSC, mesenchymal stem cell; ESC, embryonic stem cell; NSC, neural stem cell; RGL, radial glial-like neural stem cell; HFSC, hair follicle stem cell; EPC, epithelial progenitor cell; HSC, hemopoietic stem cell; hMuSC, human Muscle Stem cell; CSC, cancer stem cell; BMSC, bone marrow stromal cell; ISC, intestinal stem cell; GC, granule cell.

Stem cell interactions and their consequences include processes such as signal transmission and transduction, the response of genetic systems, and changes in stem cell behavior, which are recorded in high-resolution omics data. To date, many tools have been developed to infer cellular interactions (Nitzan et al., 2019; Browaeys et al., 2020; Cang and Nie, 2020; Efremova et al., 2020; Ren et al., 2020; Jin et al., 2021b; Dries et al., 2021; Fang et al., 2022; Li and Yang, 2022; Shao et al., 2022; Sun et al., 2022; Cang et al., 2023; Fischer et al., 2023; Tang et al., 2023). They are mainly based on scRNA-seq and spatial transcriptome data (Figure 4; Table 1).

**TABLE 1:** Tools for studying cellular interactions.

Tool	Input	Method	Roles and limitations	References
NicheNet	Gene expression data	Weighted interaction network; calculate interaction potential scores; infer signaling pathways based on scores	Analysis of target gene expression as influenced by CCC; Intracellular signaling was considered; No mention of the multisubunit structure of the ligand and receptor	Browaeys et al. (2020)
CellPhoneDB v2.0	scRNA-seq	Calculate the mean gene expression of ligand and receptor; screen for LRPs showing cell-state specificity	Subunit structures of ligands and receptors are considered; Cannot reason completely about all interactions between cells; does not take into account the spatial proximity between cells	Efremova et al. (2020)
CellChat	scRNA-seq	Identify differentially expressed signaling genes; calculate CCC probabilities; identify major signals	More suitable for predicting stronger interactions; Predicting fewer interactions	Jin et al. (2021b)
Trasig	scRNA-seq	Sort each cell of different cell clusters in proposed time and analyze their gene expression; analyze the change of gene expression with proposed time; calculate the correlation of possible ligand-receptors	Reduced false positives in CCC prediction using pseudotime analysis	Li et al. (2022b)
PAGA	scRNA-seq	Cell clustering; calculation of connectivity between cell clusters to obtain PAGA graphs; integration of PAGA graphs with databases of CCC	Based on known CCC databases	Wolf et al. (2019)
icellnet	scRNA-seq	Calculate LRP scores; visualize LRP scores	Describes how the integration of downstream signaling pathways and target gene expression profiles into CCC analysis may lead to false positive or false negative results	Noël et al. (2021)
SingleCellSignalR	scRNA-seq	LRP scoring; output LR interaction; link upstream and downstream for analysis	Based on known LRP database; based on regularized product score; ignores the spatial relationships between interacting cells	Cabello-Aguilar et al. (2020)
CellCall	scRNA-seq	Calculate communication scores and predict interactions using ligand-receptor expression and downstream TF activity	Integrates signals from inside and outside the cell; ignoring non-gene expression factors; non-protein ligand-receptor data urgently need to be supplemented	Zhang et al. (2021)
RNA-Magnet	scRNA-seq/ST	Rate the attractiveness of each cell based on the ligand-receptor expression pattern; provide the orientation of the attractor cells	the predicted attractor cell population is specific and may not be applicable to the prediction of other CCC	Baccin et al. (2020)
NATMI	scRNA-seq	Extract the expression of various ligands or receptors from different cell types; predict the interaction of cell types expressing ligands with cell types expressing homologous receptors	Ignoring information other than gene expression levels; relying on the completeness of the LRP database	Hou et al. (2020)
SoptSC	scRNA-seq	Predict the probability of signaling by the degree of ligand-receptor expression and the activity of target genes	Ignores unknown intercellular communication; not suitable for dynamic intercellular communication; analyzed intercellular communication is unidirectional	Wang et al. (2019)
PIC-seq	scRNA-seq	sequences PIC to obtain scRNA-seq data, combined with single-cell sequencing data to deconvolute the PIC complex into several single cells	Identify intracellular physical interactions; based on cell multiplets	Giladi et al. (2020)
Neighbor-seq	scRNA-seq	Creating artificial multiplets; machine learning to distinguish between different combinations of multiplets; evaluating multiplet enrichment; calculating enrichment scores for CCI; constructing CCI networks	based on cell multiplets	Ghaddar and De (2022)
CIM-seq	scRNA-seq	Retention of cell multiplets in single cell suspensions for RNA sequencing; deconvolution of transcriptome data to break up cell multiplets; differential gene expression analysis		Andrews et al. (2021)
Tools based on ST				
COMMOT	ST and scRNA-seq	Optimal transport strategies were used to analyze the direction and strength of CCC	Visualizing CCC, annotating CCC direction, and analyzing CCC downstream effect; The possibility of a false positive of CCC; Neglect of *in situ* space proximity	Cang et al. (2023)
NCEM	scRNA-seq; MERFISH data	The results of ST data analysis were used as input information to train NCEM to predict the spatial specificity of cellular gene expression	Based on GNN; Reasoning about intercellular communication and simulating ecological niche effects	Fischer et al. (2023)
SpaOTsc	scST/scRNA-seq with corresponding spatial data	The scRNA-seq data were corroborated with spatial transcriptome data; structured optimal transport strategy was used	Reconstructing intercellular communication and estimating the spatial characteristics of intercellular signals; the judgment of cell interactions is based on the spatial distance between cells, and may not be suitable for all types of cell interactions	Cang and Nie (2020)
SpaCI	scST	Projecting cell spatial location and gene expression patterns into the same latent space; using a triplet loss training model to determine whether an LRP interacts with each other	Revealing the relationship between TFs and ligands and the ligand-receptor pair	Tang et al. (2023)
STRIDE	scRNA-seq; ST	Decomposition of spatial transcriptome data into components containing spatial information and other components, and integration with scRNA seq data	Inferring intercellular interactions; A high match of spatial data with scRNA-seq data is required; Insufficient ability to discriminate between similar cell types within the same lineage	Sun et al. (2022)
SpaTalk	single-cell and spot-based ST	GNN learning is used to calculate LRI probabilities; the most likely cellular interactions are filtered by combining LRI probabilities with spatial distances	Inferring cellular communication and signaling pathways; Deficiencies in inferring cellular telecommunications; the LRP database maybe incomplete	Shao et al. (2022)
Giotto	10X Genomics Vissium data	Cell clustering; Genes with spatial differential expression or correlation were searched	visualizing spatial histology data; Interactions between cell types rather than between cells are analyzed	Dries et al. (2021)
DeepLinc	Deep learning from cell interactions and ST	Cell interactions are directly learned from scST data using VGAE, skipping cell clustering	Discovery of new cell types; Reconstruct a complete cell interaction landscape; Inferring cellular telecommunications; Only single cell spatial transcriptome data can be used and dynamic cellular interactions, such as cell differentiation, cannot be analyzed	Li and Yang (2022)

ST, spatial transcriptomics; scST, single cell spatial transcriptomics; TF, transcription factor; LRTF, ligand-receptor-TF axis; LRI, ligand-receptor interaction; LRP, ligand-receptor pairs; LRT, ligand-receptor-target; CCC, cell-cell communication; FDC, follicular dendritic cell; CAF, cancer-associated fibroblast; GNN, graph neural network; NCEM, node-centric expression model; PIC, physically interacting cells; VGAE, variational graph autoencoder.

### 5.1 Single-cell RNA sequencing (scRNA-seq)

scRNA-seq is a mighty method for analyzing information regarding intra- and extracellular interactions using whole transcriptional profiling (Travaglini et al., 2020; Wang et al., 2020; Wang et al., 2021a). scRNA-seq is mainly used to identify different cell types and map the developmental trajectory of cells (Paik et al., 2020). In addition, scRNA-seq can be used to predict cell interactions. scRNA-seq data are used to perform computer modeling to build tissue-level models to predict intercellular interactions through ligand-receptor pairs (Del Sol and Jung, 2021).

To systematically use transcriptome data to infer cell-cell interactions and generate potential cell-cell communication networks, many analytical tools have been developed, including NicheNet, CellPhoneDB and CellChat (Vento-Tormo et al., 2018; Browaeys et al., 2020; Centonze et al., 2020; Efremova et al., 2020; Jin et al., 2021b). These considerations have been updated using newer technologies from only one ligand-receptor gene pair used to the integrated consideration of different subunit states in receptors as multi-subunit complexes (Efremova et al., 2020). Moreover, more complete data on cellular interactions were compiled (Jin et al., 2021b). Important signaling cofactors and cellular spatial locations have also been considered (Efremova et al., 2020; Saviano et al., 2020; Jin et al., 2021b). Compared to CellPhoneDB, NicheNet can also analyze the gene expression of receiver cells (Browaeys et al., 2020). CellChat performs better in predicting strong intercellular interactions (Jin et al., 2021b). Furthermore, the developers of CellChat described the application of the tool in the pseudotime analysis of continuous cell states. But the systematic description of the application of the pseudotime analysis in improving intercellular interactions was not reflected until the development of TraSig (Li et al., 2022b). TraSig focuses on the temporal heterogeneity of gene expression in homogeneous clusters of cells by including ligand-receptor genes that are expressed at similar rates in the pseudotime in the prediction criteria for positive ligand-receptor pair interactions.

Table 1 compares in detail the differences between tools for analyzing cell-to-cell interactions using transcriptome data, including tools mentioned above. There are concerns that distant endocrine signals are difficult to capture owing to the technical limitations of scRNA-seq (Liu et al., 2022b; Dimitrov et al., 2022). CCI mediators have also been studied in a relatively homogeneous manner, and attempts have been made to investigate mediators other than proteins (Dimitrov et al., 2022).

### 5.2 Spatial transcriptomics (ST)

ST technologies discussed in this paper refer to technologies aimed at preserving spatial information and obtaining transcriptome information (Ståhl et al., 2016). ST provides spatial information and is used to study cell-cell contact. The technical shortcomings of ST are reflected in the lack of resolution and transcriptome coverage, but these shortcomings are being rapidly filled (Longo et al., 2021; Rao et al., 2021; Dimitrov et al., 2022; Tian et al., 2022). The ability of spatial transcriptomics to reveal the identity, extent, and spatial location of expressed genes (Kobayashi et al., 2020) links tissue biology to transcriptomics and is a powerful means of exploring the local representation of spatial patterns of cellular gene expression. For example, based on single-cell analysis of ST, Kadur Lakshminarasimha Murthy et al. (2022) revealed the fate trajectory of a bipotent progenitor cell population in the lungs during lung regeneration. This is different from the process that occurs in the lungs of mice (Choi et al., 2020; Kobayashi et al., 2020). Visual analysis based on ST has depicted the entire process from human pluripotent stem cells to hematopoietic stem cells (Calvanese et al., 2022), validating and gaining a deeper understanding of previous studies (Zhou et al., 2016). The current description of *in situ* cellular interactions can be achieved by sorting physically interacting cell groups using scRNA-seq (Giladi et al., 2020). This technique overcomes the limitation of missing spatial information when using scRNA-seq alone to analyze the expression of intercellular contact-dependent genes (Kim et al., 2023). An application has also been developed for the analysis of physical interactions in complex tissues, using unsupervised and high-throughput multiplex sequencing techniques that can reconstruct the spatial structure of the interactome (Andrews et al., 2021). Furthermore, the identification and labeling of intercellular contacts and ligand-receptor signaling from massively parallel single-cell sequencing data have been achieved (Ghaddar and De, 2022). Quantification of cell-cell interactions can be achieved using *in situ* molecular colocalization. Its advantage over chromatin immunoprecipitation sequencing or ribosome analysis is the ability to perform *in situ* analyses (Tian et al., 2022). Moreover, a technique has been proposed for human clinical samples without the use of genetic modification strategies, which provides sample transcript information and long-term, stable tracking of cytodynamics. It can be used as a complement to spatial omics and typical single-cell sequencing (Genshaft et al., 2021). The use of cell space maps has been proposed to improve the accuracy of inference of intercellular communication (Fischer et al., 2023). Mathematical models are developed centered on nodes to simulate ligand-receptor interactions to explain intercellular interactions and reduce the rate of misinterpretation of intercellular dependence. A recent technique based on collective optimal transport, COMMOT, was proposed for inferring and visualizing intercellular communication (Cang et al., 2023). However, the authors pointed out the possibility of false positives in the results generated by applying this technique. In addition, Zhu et al. (2023) developed a platform for simulating real ST data, which provides an efficient and low-cost data source for evaluating different spatially resolved transcriptomics techniques.

## 6 Update on the stem cell interaction mechanism theory

Adult tissue-specific stem cells receive biochemical or mechanical signals from cellular or non-cellular components of the niche, generating a series of intracellular signal transduction pathways, which in turn undergo structural or functional changes (Pinho and Frenette, 2019; Brunet et al., 2023). Biochemical signaling between cells may occur in three forms: 1) free diffusion of ligands, which includes both autocrine and paracrine mechanisms; 2) ligands are secreted and present in the extracellular matrix (ECM), and receptors bind to these ligands when cells come into contact with the ECM; 3) ligands are expressed on the cell surface, and cells expressing surface receptors and cells expressing surface ligands are transmitted through direct binding of ligand-receptor signals. In contrast, mechanical information transmission between cells is mainly related to intercellular connections and the ECM nature and structure (Humphrey et al., 2014).

### 6.1 Free diffusion

#### 6.1.1 Autocrine

Initially, cellular autocrine was more often studied using cancer cells as a model, and this mechanism was found to control the growth of various cells, especially to stimulate cell proliferation (Adkins et al., 1984; Huang et al., 1984; Lang et al., 1985; Sporn and Roberts, 1985). The ability of the autocrine mechanism to stimulate cell proliferation has also been demonstrated in normal tissues under physiological conditions as well as in stem cells (Gaudio et al., 2006; Abdel-Malak et al., 2008; Gallipoli et al., 2013; Pardo-Saganta et al., 2015). Autocrine signaling, an important stem cell communication mechanism, has been studied extensively. Pathways closely related to stem cell autocrine such as TGF-β, VEGF, mTOR, SHH, and Wnt signaling pathways have been revealed (Lim et al., 2013; Chen et al., 2014; Hsu et al., 2014; Nabhan et al., 2018; Yeh et al., 2018; Zhou et al., 2018; Morinaga et al., 2021).

In the lung, alveolar type 2 cells (AT2) act as alveolar adult tissue-specific stem cells in response to injury (Nabhan et al., 2018; Zacharias et al., 2018). AT2 cells secrete Wnt to recruit more AT2 cells and prevent daughter cells from undergoing transdifferentiation. Axin2+ cells in the skin contribute significantly to wound healing, and its proliferation requires Wnt/β-catenin signaling activation (Lim et al., 2013). In turn, Axin2+ cells themselves can secrete Wnt and self-renew through an autocrine mechanism. Sustained neurogenesis in adult neural stem cells requires autocrine Mfge8 signaling (Zhou et al., 2018). Zhou et al. (2018) used single-cell transcriptome analysis to identify Mfge8 transcripts in resting radial glial-like neural stem cells (RGL). Mechanistically, Mfge8 enrichment inhibited mTOR1 signaling and prevented RGL overactivation and depletion. Additionally, RGL can autocrinologically produce endothelin-1 to promote their proliferation and maintenance (Adams et al., 2020). It has been demonstrated that maintaining transit-expanded cell populations requires autocrine SHH production, which is also necessary for the proliferation of hair follicle stem cells (Hsu et al., 2014). Morinaga et al. (2021) found that autocrine/paracrine IL-1R resulting from a high-fat diet was associated with NK-kB activation and SHH inhibition, depleting hair follicle stem cells and macroscopically accelerating hair loss. Temporal heterogeneity of thymic epithelial types was revealed by scRNA-seq, which was combined with CRISPR-Cas9 technology to characterize the changes in the thymic epithelium over time in more detail (Nusser et al., 2022). This experiment also showed that autocrine secretion of Fgf7 continues to stimulate massive proliferation of the thymic epithelium, but the pool of epithelial progenitor cells is not depleted, and the characteristics of the thymic epithelium remain unchanged (Nusser et al., 2022). In bone tissue models, the production of tumor necrosis factor (TNF-α) released by MSCs is thought to be a key factor in the maintenance of self-renewal and differentiation of MSCs and their involvement in maintaining bone homeostasis (Yu et al., 2021). Similar results were observed for jawbones. The expression and secretion of PPR by PTHrP+ MSCs in dental follicles are necessary for maintaining a normal cell fate (Takahashi et al., 2019). During embryogenesis, embryonic stem cells (ESCs) produce a cytokine-containing Wnt receptor that allows ESCs to distinguish between niche signals (Junyent et al., 2020). ESCs actively select Wnt ligands secreted by trophectodermal stem cells to promote self-renewal and synthesize more cytokines, thereby participating in accelerated embryogenesis. Post-hematopoietic stem/progenitor cells also benefit from autocrine signaling, and the mechanisms involved are the PI3K/AKT, MAPK/ERK, JAK/STAT, and NK-kB signaling pathways, which have been sorted out (Hurwitz et al., 2020; Stone et al., 2022).

#### 6.1.2 Paracrine

Most paracrine studies on stem cells focus on the secretome of MSCs (Chang et al., 2021). This regenerative medicine branch does not focus on directly exploiting the proliferative and differentiation capacity of stem cells but rather on the regenerative and immunomodulatory potential of MSCs secretions (Kumar et al., 2019). It is worth clarifying that mesenchymal stem cell (MSC) is recently not considered a “stem cell” precisely because they lack multipotency *in vivo* (Caplan, 2017). The use of medicinal signaling cells as the true meaning of MSCs has gained some acceptance (de Windt et al., 2017).

Proteomics-based analyses have shown that stem cell secretomes from different ecological niches have different functions (Kumar et al., 2019). For different application contexts, we can use different tailored secretomes or even factors such as biomaterials to control efficacy (Chang et al., 2021). Given that in recent years there has been dedicated literature to identify various MSC secretome delivery options, this paper will not repeat them in this section (Chang et al., 2021; Han et al., 2022).

Chemical molecules secreted by non-stem cells have powerful and multi-effect regulatory effects on the biological behavior of stem cells. T cells in the brains of older individuals inhibit neural stem cell proliferation by secreting interferon γ (Dulken et al., 2019).

### 6.2 Proximity communication between cells

Interactions between cells nearby may occur by passing certain cellular substances or ligand-receptor binding. In this subsection, intercellular connections transmit biochemical signals, which are distinguished from mechanical signals in the next section.

#### 6.2.1 Embryonic developmental stage

During embryonic development, a positive feedback loop exists between the length of intercellular contacts and the nodal signaling pathway (Barone et al., 2017). This positive feedback loop controls decisions regarding the fate of the developing embryos. Furthermore, intercellular contacts between posterior lateral plate mesodermal cells and somitic cells expressing Notch ligands allow the former to acquire the identity of a hematopoietic stem cell precursor, that is, blood-derived endothelium (Rho et al., 2019).

#### 6.2.2 Nervous system

Experimental nervous system models illustrate that ephrin-B3 downregulation on the cell membrane of excited hippocampal dentate granule cells (GCs) triggers EphB2 signaling attenuation in adjacent radial neural stem cells (rNSCs) through direct cell contact, leading to rNSC activation and the generation of new neurons (Dong et al., 2019). Conversely, when enhanced EphB2-ephrin-B3 signaling contributes to the maintenance of the quiescent state of rNSCs. In contrast, intercellular contacts affect gene expression and fate determination in human-induced pluripotent neural stem/progenitor cells cultured *in vitro* (McIntyre et al., 2022). Transcriptome analysis identified the differential expression of Notch and Wnt, further suggesting that Notch and Wnt are responsible for the neurogenic cell fate of neural stem/progenitor cells.

#### 6.2.3 Immune system

##### 6.2.3.1 The biological behavior of stem cells is modulated

Interactions between immune cells and stem cells have also been reported. The fate of intestinal stem cells (ISC) is regulated by adhesion signals from immune cells (Chen et al., 2021). Binding of integrin αEβ7 expressed by T cells to E-cadherin on ISC cell membranes triggers Wnt signaling promotion and inhibition of Notch signaling, thus maintaining normal ISC differentiation. Biton et al. detected MHC II enrichment in Lgr5 ISC using scRNA-seq, revealing the influence of the interactions between Th cells and Lgr5 ISC on stem cell renewal and differentiation. Pro-inflammatory signals can promote intestinal stem cell differentiation (Biton et al., 2018). The mechanism of aplastic anemia is also related to the interaction between T cells and hematopoietic stem/progenitor cells, leading to their destruction (Zhu et al., 2021).

The paracrine effects of stem cells are also influenced by the cell-cell contact between stem cells and other cells. For example, the immunomodulatory effects of MSCs are enhanced upon contact with pro-inflammatory macrophages (Li et al., 2019). Additionally, macrophages can direct muscle stem cells to repair skeletal muscles by secreting stem cell niche signals (Ratnayake et al., 2021). Vascular cells and lymphatic vessel cells can be considered as components of stem cell niche (Kusumbe et al., 2014; Itkin et al., 2016; Biswas et al., 2023). Lymphatic vessels influence the proliferation and differentiation of hemopoietic stem cells and bone progenitor cells through the secretion of CXCL12.

##### 6.2.3.2 The biological behavior of cells in contact with stem cells is modulated

Moreover, immune cell function is affected by intercellular contact between stem cells and immune cells. This may be due to the secretion of cytokines that affect stem cells (Alunno et al., 2018). There are other cases; for example, the transfer of active mitochondrial and plasma membrane fragments to Tregs occurs during intercellular contact between MSCs and Treg cells dependent on HLA, allowing for enhanced Treg immunosuppression (Piekarska et al., 2022). MSCs enhance Treg immunosuppression through contact-dependent interactions that are partly mediated by MSC-expressed CD80 (Mittal et al., 2022). Myogenic hMuStem cells suppress T cell activity and promote Treg production through paracrine or intercellular contacts (Charrier et al., 2022). Thus, hMuStem cells are potent immunomodulators. Particularly, cancer-initiation stem cells expressing CD4 inhibit the activity of cytotoxic T cells through direct contact with the latter (Miao et al., 2019).

Stem cells can also regulate immune cell functions through paracrine and intercellular contacts (Lynch et al., 2020). TGF-β MSC enhanced immune suppression is Smad2/3 dependent as well as intercellular contact-dependent. In contrast, TGF-β MSC promotes Treg expansion and T cell activation through paracrine secretion when PGE2 is the main mediator.

#### 6.2.4 Bone

In bone marrow tissue, bone marrow regeneration after radiation clearance is inseparable from the transfer of mitochondria from hematopoietic stem cells to bone marrow mesenchymal stromal cells through intercellular contacts (Golan et al., 2020). Hematopoietic stem/progenitor cells are extensively polarised after contact with bone marrow stromal cells specifically via SDF1 (Bessy et al., 2021). Specific interactions between developing neutrophils and megakaryocytes are also observed in the bone marrow (Boisset et al., 2018). Their interaction is enhanced under certain pathological conditions because neutrophils can survive inside megakaryocytes. This study also revealed an interaction between Lgr5+ stem cells and Tac1+ enteroendocrine cells. The experiment leaves room for improvement in judging whether the interaction occurs or not, as the authors mention that “the expected interaction needs to be verified *in situ*.”

### 6.3 Mechanical regulation

#### 6.3.1 Perception and transmission of mechanical information by stem cells

Mechanical signals include both the structure and properties of the ECM and forces generated by the cell through various connections (Humphrey et al., 2014). This information is translated through mechanotransduction into information that affects gene expression, linking upstream and downstream biological responses (Zanconato et al., 2016; Panciera et al., 2017). Mechanotransduction initiation is associated with the recognition of external mechanical information by various membrane proteins, such as cell-expressed adhesion molecules, and the feedback of mechanical forces by the cytoskeletal structure within the cell (Totaro et al., 2018). Recent studies revealed the contribution of transcription factors Yes-associated protein (YAP) and transcriptional coactivator with PDZ-binding motif (TAZ) to mechanical information input (Meng et al., 2018; Totaro et al., 2018; Zanconato et al., 2019). The work of Totaro et al. (2018) showed that the ECM-integrin-, F-actin-, Hippo-, Wnt-, and G protein-coupled receptor-YAP/TAZ pathways, providing essential information on how YAP/TAZ acts as a transcription factor in response to upstream signals and regulates downstream pathways. Mechanoreceptive information via focal adhesions is the main source of information received by YAP/TAZ (Vining and Mooney, 2017). Wang et al. (2021b) identified cerebral cavernous malformation 3 (CCM3), an upstream molecule that regulates YAP/TAZ, and demonstrated that MSC differentiation is influenced by this pathway. CCM3 is localized at focal adhesion sites in cancer-associated fibroblasts and MSCs and controls mechanotransduction and YAP/TAZ activity. Meng et al. (2018) identified another upstream molecule of the Hippo pathway, Ras-related GTPase 2(RAP2), which mediates the ECM rigidity-YAP/TAZ-nucleus signaling pathway. RAP2 specifically transmits ECM rigidity signals and inhibits YAP/TAZ through a series of reactions after activation under low-rigidity conditions. Chang et al. (2018) complemented the inhibitory effect of the SWI/SNF complex on YAP/TAZ and proposed that increased nuclear YAP/TAZ accumulation and SWI/SNF complex inhibition were two necessary conditions for obtaining a YAP/TAZ response. In addition, two actin cytoskeleton regulators upstream of YAP1 have been reported (Aragona et al., 2020). Changes in gap junctions in the human papilla stem can also mediate stem cell interactions with the external physical microenvironment (Zhou et al., 2020). Primary cilia are also involved in the mechanical regulation of human tissues and can play a role in promoting the differentiation of different stem cell populations (Chen et al., 2016; Li et al., 2022c; Palla et al., 2022).

#### 6.3.2 Mechanically informed stem cell self-renewal regulation and proliferation

Mechanically gated Piezo1 channels are expressed in both neural stem cells and astrocytes and regulate adult neurogenesis (Pathak et al., 2014; Chi et al., 2022). In *Drosophila*, the proliferation and differentiation of all stem cells that ectopically express Piezo are promoted by a mechanism that is inseparable from calcium signaling (He et al., 2018). Piezo1 is also expressed in muscle stem cells (MuSCs), where it transmits mechanical signals that help maintain MuSC quiescence and prevent senescence (Peng et al., 2022). Furthermore, Piezo1 mediates changes in MuSC status, which promotes skeletal muscle regeneration (Ma et al., 2022). During increased muscle loading, MSCs promote muscle stem cell proliferation through the Yap1/Taz-Thbs1-CD47 pathway (Kaneshige et al., 2022). When there is muscle sclerosis due to injury, etc., this change in physical information is transmitted to the nucleus via YAP/TAZ to maintain muscle stem cell activation and proliferation (Silver et al., 2021). Adult muscle stem cells maintain quiescence through the interaction of calcitonin receptors with secreted collagen (Baghdadi et al., 2018). The Notch–collagen V-calcitonin receptor signaling cascade may play similar roles in different stem cell populations.

Wnt and Src-YAP signaling cooperate to drive intestinal regeneration (Guillermin et al., 2021). Additionally, intracellular crowding due to compressive mechanical information enhances Wnt/β-catenin signaling and promotes ISC self-renewal (Li et al., 2021).

#### 6.3.3 Mechanical information regulates the fate decision of stem cells

Physical signaling with tunable properties that act immediately and locally is an object of interest to regulate the differentiation of stem cells into specific lineages (Kong et al., 2021). The ECM-integrin α5-F-actin-YAP1-Notch signaling way regulates the fate decisions of bipotent pancreatic progenitor cells, especially the first half, i.e., the integrity of extracellular matrix-integrin α5 promotes ductal lineage fate and *vice versa* in favor of endocrine cell fate (Mamidi et al., 2018). MSCs are often used in bone regeneration engineering. The mechanical signal of reduced collagen alignment in the ECM is captured by MSCs, causing MSCs to prefer the adipose to osteogenic fate (Huber et al., 2020). Smaller differences in mechanical force information can determine whether MSCs choose an osteogenic or lipogenic fate via the myosin II, Rac1, Src, FAK, YAP, and TAZ signaling pathways (Han et al., 2019). The integrin/N-calmucin-cofilin-actin-YAP pathway leads to the osteogenic differentiation of human MSCs (Zhang et al., 2022b). Moreover, osteogenic differentiation of periodontal ligament stem cells can be promoted by enhanced Tet2/HDAC1/E-calmodulin/β-linked protein signaling (Yu et al., 2022b). The intercalated disc-mediated mechanosensory pathway between cardiac myocytes eventually activates the ectopic adipogenic program, which partly explains why cardiac adipocytes share a common precursor with some cardiac myocytes (Dorn et al., 2018).

Indeed, numerous researchers have shed a light on how mechanical information affects cell behavior. Chaudhuri et al. (2020) previously focused on the effects of the ECM on cell behavior, providing insights. Kong et al. (2021) elucidated the mechanisms by which nanomaterials applied in stem cell research transmit physical signals that affect the fate of stem cells. Valet et al. (2022) focused on the vertebrate embryogenesis stage and described how mechanical signaling regulates key developmental processes during this period. A recent study systematically organized the mechanisms by which biomechanics regulate cell fate, with a focus on mechanical transduction, intracellular signaling, and cell surface mechanics (De Belly et al., 2022). Methods and tools with higher spatial and temporal resolutions are still required.

## 7 Overview and perspectives

We are currently studying the mechanisms underlying stem cell interactions to improve the application of stem cells in regenerative medicine. To systematically and precisely elucidate these mechanisms, technologies with higher spatial and temporal resolutions, more realistic simulations of the stem cell microenvironment, and more specific stem cell population differentiation and tracing are required. This demand has led to many powerful technologies that elucidate the mechanisms of stem cell interactions. We discuss how lineage tracing, synthetic receptors, proximity labeling and transcriptome data analysis tools can be applied in the study of CCI. The techniques presented in this paper include but are not limited to using stem cells as research models, and using non-stem cells as research models has complementary and reference significance. In addition, we note some other techniques. For example, SPEAC-seq based on CRISPR-Cas9 with microfluidic microarrays to identify cell signaling pathways (Wheeler et al., 2023). It provides a demonstration of CRISPR-Cas9 applied to CCI studies.

The study of stem cell interactions is in a rapidly evolving stage, especially in the context of rapid iterative updates in biotechnology. Although, the biological understanding of stem cell interactions sorted out in this paper is not sufficient to elaborate a comprehensive picture of stem cell behavior during mammalian development or injury repair, it contributes to a complete description of stem cell interaction mechanisms in the future.
